# Supplementary material for: Basal ganglia and cerebellum contributions to vocal emotion processing as revealed by high-resolution fMRI
Source: Sci Rep. 2021 May 20;11:10645. doi: 10.1038/s41598-021-90222-6 (PMC8138027; doi:10.1038/s41598-021-90222-6)
Supplement: Supplementary file 1 — Supplementary Information. [file 41598_2021_90222_MOESM1_ESM.pdf]

Basal ganglia and cerebellum contributions to vocal emotion processing as revealed by high-resolution fMRI

Leonardo Ceravolo[1,2*], Sascha Frühholz[3,4,5], Jordan Pierce[6], Didier Grandjean[1,2‡], & Julie Péron[6,7‡]

‡ *These authors contributed equally to this work*

[1] Neuroscience of Emotion and Affective Dynamics laboratory, Department of Psychology and Educational Sciences

[2] Swiss Centre for Affective Sciences, University of Geneva, Switzerland

[3] Department of Psychology, University of Zürich, Zürich, Switzerland

[4] Neuroscience Center Zurich, University of Zurich and ETH Zurich, Zurich, Switzerland

[5] Department of Psychology, University of Oslo, Oslo, Norway

[6] Clinical and Experimental Neuropsychology Laboratory, Department of Psychology and Educational Sciences, University of Geneva, Switzerland

[7] Cognitive Neurology Unit, Department of Neurology, University Hospitals of Geneva, Geneva, Switzerland

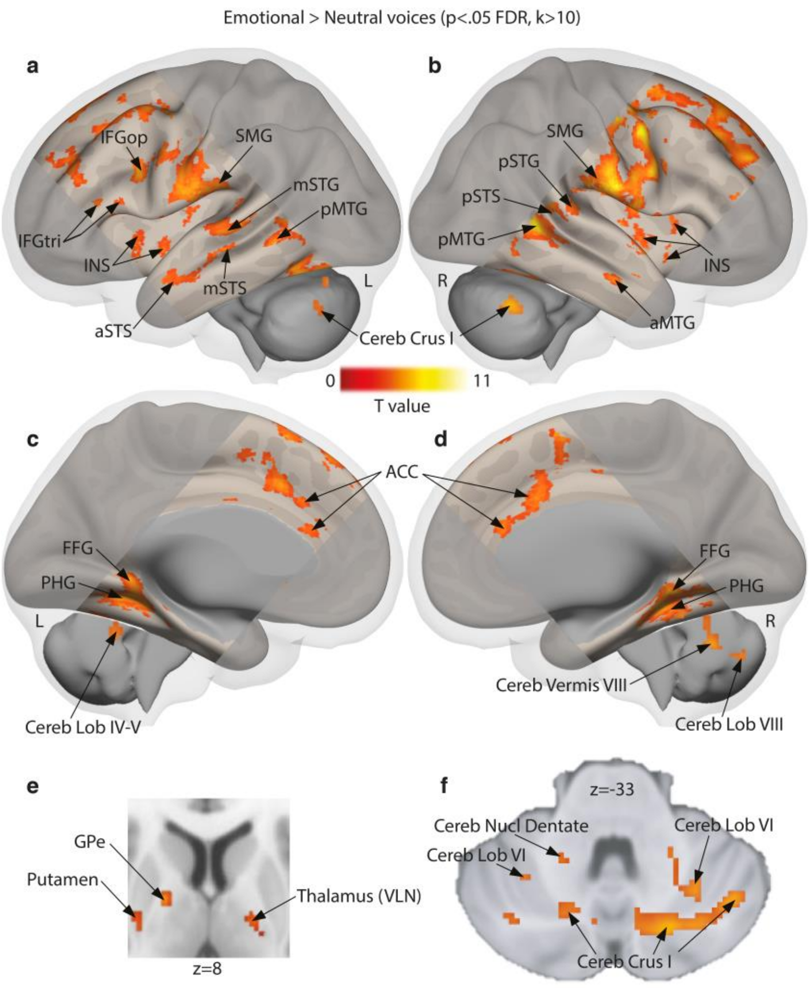

Fig.S1: Brain measures for implicitly processing angry and happy compared to neutral voices, corrected for multiple comparisons (whole brain voxelwise *p*<.05 FDR, k>10 voxels). A-B Lateral activations rendered on a sagittal image highlighting middle and superior temporal regions. C-D Medial activations of the anterior cingulate cortex, parahippocampal cortex and cerebellum. E Subcortical activity in the thalamus and globus pallidus displayed on an axial slice. F Cerebellar activations displayed on an axial slice. White outline: sample-specific temporal voice areas, thresholded at whole brain voxelwise *p*<.05 FDR corrected at the voxel level. L: left; R: right; IFGop: inferior frontal gyrus pars opercularis; IFGtri: inferior frontal gyrus pars triangularis; STG: superior temporal gyrus; STS: superior temporal sulcus; MTG: middle temporal gyrus; STS: superior temporal sulcus; INS: insula; SMG: supramarginal gyrus; FG: frontal gyrus; FFG: fusiform gyrus; PHG: parahippocampal gyrus; ACC: anterior cingulate cortex; Cereb: cerebellum; Cereb Lob: cerebellum lobule; Cereb Nucl Dentate: dentate nucleus of the cerebellum; Thalamus VLN: ventral lateral nucleus of the thalamus; GPe: external globus pallidus; Cereb Crus: cerebellum crus of ansiform lobule. 'a' prefix: anterior part; 'm' prefix: mid part; 'p' prefix: posterior part.

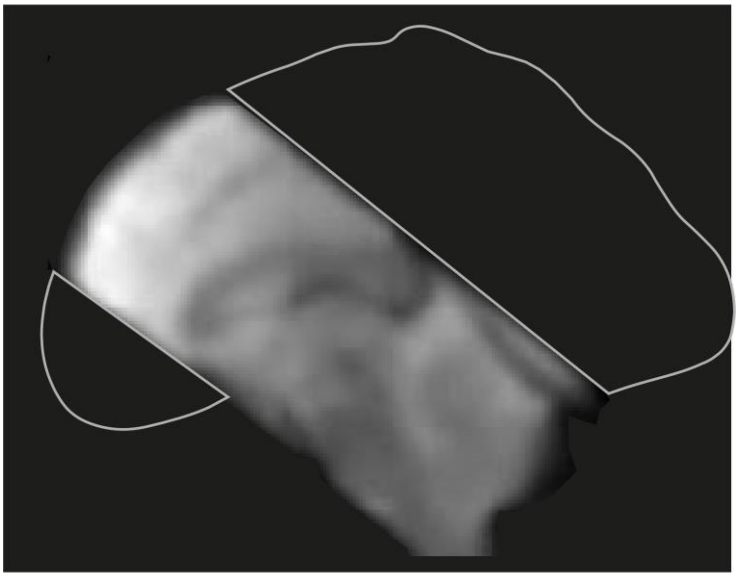

Fig.S2: Illustration of the truncated field of view of the high-resolution functional MRI scans acquired continuously during the one-back task.

Supplementary Table 1: Activations, cluster size and coordinates for normal voices>F0 flattened voices & voice mean energy contrast, whole brain voxelwise $p<.05$ FDR correction, k>10.

		MNI coordinates				
Region label	Hemisphere	X	Y	Z	T value	Cluster size (voxels)
STG, posterior	R	54	-34	12	14.27	7688
Precentral gyrus	*R*	*60*	*8*	*26*	*13.35*	
ITG, posterior	L	-46	-36	-16	9.43	198
ITG, posterior	*L*	*-44*	*-54*	*-12*	*4.29*	
ITG, posterior	*L*	*-52*	*-44*	*-16*	*4.29*	
Cereb Lob 6	R	20	-56	-16	7.93	356
Fusiform gyrus	*R*	*34*	*-62*	*-20*	*6.82*	
Cereb Lob 6	*R*	*30*	*-60*	*-28*	*6.10*	
Mid Frontal gyrus	R	36	26	20	7.23	994
Mid Frontal gyrus	*R*	*24*	*28*	*36*	*7.12*	
Mid Frontal gyrus	*R*	*28*	*38*	*28*	*7.01*	
STS, posterior	R	60	-46	4	7.12	37
MTG, anterior	R	66	-4	-16	6.90	60
Mid Frontal gyrus	L	-38	34	20	6.73	376
IFG triangularis	*L*	*-52*	*30*	*12*	*5.09*	
Mid Frontal gyrus	*L*	*-32*	*38*	*24*	*5.05*	
IFG opercularis	L	-50	10	8	6.45	53
IFG opercularis	*L*	*-58*	*14*	*8*	*4.69*	
Supp Motor tracts Area	R	10	6	58	5.87	34
Hippocampus, anterior	L	-26	-6	-24	5.37	31
Supp Motor tracts Area	L	-2	22	60	5.21	169
Cingulate cortex	R	10	14	42	5.18	43
Cereb Lob 8	R	20	-70	-44	5.11	64
Cereb Crus 2	R	8	-82	-36	5.05	24
Hippocampus, anterior	L	-12	-10	-22	4.92	38
Cereb Lob 8	L	-18	-58	-20	4.87	135
Cereb Nucl Dentate	R	14	-48	-28	4.75	14
STG, mid	R	66	-16	6	4.72	22
IFG, triangularis	L	-42	18	6	4.68	37
Sup Frontal gyrus, medial	R	12	50	34	4.61	42
Cereb Crus 1	L	-38	-80	-30	4.52	14
Putamen	L	-16	8	-12	4.43	19
Cereb Lob 8	L	-28	-58	-52	4.43	20
Cereb Vermis 4-5	L	-2	-58	-26	4.35	47
Sup Frontal gyrus	L	-14	36	48	4.25	50
ACC	R	6	16	28	4.23	26

Sup Frontal gyrus, medial	L	-8	48	34	4.22	38
ACC	R	2	34	20	4.05	39
Thalamus	L	-18	-18	12	4.02	37
Globus Pallidus	R	18	-4	10	2.96	11

L: left; R: right; MNI: Montreal neurological institute; STG: superior temporal gyrus; ITG: inferior temporal gyrus; Cereb Lob: cerebellum lobule; Mid: middle; STS: superior temporal sulcus; MTG: middle temporal gyrus; IFG: inferior frontal gyrus; Supp Motor tracts Area: supplementary motor tracts area; Cereb Crus: cerebellum crus of ansiform lobule; Cereb Nucl Dentate: dentate nucleus of the cerebellum; Sup: superior; Cereb: cerebellum; ACC: anterior cingulate cortex.

Supplementary Table 2: Activations, cluster size and coordinates for F0 flattened voices>normal voices & voice mean energy contrast, whole brain voxelwise *p*<.05 FDR correction, k>10.

Region label	Hemisphere	MNI coordinates X	Y	Z	T value	Cluster size (voxels)
STG, anterior	L	-62	-6	4	19.03	12010
IFG triangularis	*R*	*58*	*22*	*6*	*16.42*	
STG, posterior	*L*	*-40*	*-24*	*2*	*15.51*	
IFG opercularis	*L*	*-60*	*6*	*14*	*13.99*	
IFG opercularis	*R*	*64*	*6*	*14*	*11.92*	
STG, mid	*L*	*-48*	*-18*	*2*	*11.91*	
MTG, anterior	*R*	*60*	*-10*	*-12*	*11.88*	
Precentral gyrus	*L*	*-62*	*4*	*20*	*11.38*	
STS, mid	*R*	*60*	*-20*	*-6*	*11.09*	
MTG, anterior	*R*	*58*	*-6*	*-14*	*10.55*	
Supp Motor tracts Area	R	8	14	52	14.98	2525
Sup Frontal gyrus, medial	*R*	*4*	*40*	*54*	*9.07*	
Cereb Crus 2	R	16	-78	-40	9.04	109
Cereb Lob 6	R	18	-72	-24	7.90	123
ITG, posterior	R	46	-60	-12	7.76	267
Cereb Lob 7b	L	-30	-70	-48	7.06	148
Cereb Lob 8	R	34	-62	-54	6.65	102
Cereb Crus 2	L	-14	-82	-40	6.51	232
Cereb Crus 1	*L*	*-20*	*-76*	*-24*	*4.70*	
Cereb Lob 6	L	-34	-66	-26	6.28	96
Cereb Vermis 9	L	0	-60	-38	6.23	504
Cereb Lob 9	*R*	*12*	*-46*	*-48*	*6.22*	
Cereb Lob 9	*R*	*14*	*-62*	*-46*	*4.82*	
Cereb Crus 1	L	-40	-52	-40	6.13	20
Cereb Lob 4-5	L	-6	-48	-14	5.20	52
ITG, posterior	L	-52	-62	-10	4.51	27
IFG triangularis	L	-50	36	26	4.28	26
Cereb Crus 1	L	-46	-62	-30	3.76	13

L: left; R: right; MNI: Montreal neurological institute; STG: superior temporal gyrus; IFG: inferior frontal gyrus; MTG: middle temporal gyrus; Supp Motor tracts Area: supplementary motor tracts area; Sup: superior; Cereb Crus: cerebellum crus of ansiform lobule; Cereb Lob: cerebellum lobule; Cereb: cerebellum;; ITG: inferior temporal gyrus.

Supplementary Table 3: Activations, cluster size and coordinates for voice mean energy> normal & F0 flattened voices contrast, whole brain voxelwise $p<.05$ FDR correction, k>10.

Region label	Hemisphere	X	Y	Z	T value	Cluster size (voxels)
		MNI coordinates				
MTG, posterior	L	-68	-36	-6	13.54	23425
Supramarginal gyrus	*R*	*56*	*-2*	*42*	*12.24*	
STS, posterior	*L*	*-46*	*-42*	*2*	*10.93*	
MTG, posterior	*R*	*54*	*-34*	*-8*	*10.70*	
Putamen	*R*	*28*	*-10*	*10*	*10.07*	
STS, posterior	*R*	*70*	*-34*	*4*	*10.05*	
MTG, posterior	*R*	*68*	*-38*	*4*	*10.04*	
MTG, posterior	*R*	*66*	*-42*	*6*	*9.98*	
Rolandic operculum	*R*	*48*	*-2*	*12*	*9.25*	
Cereb Crus 1	*R*	*20*	*-70*	*-36*	*8.59*	
Cereb Crus 1	*L*	*-14*	*-68*	*-30*	*8.37*	
Cereb Crus 1	*L*	*-22*	*-72*	*-30*	*8.34*	
IFG opercularis	L	-60	12	28	7.45	516
Precentral gyrus	*L*	*-58*	*2*	*34*	*7.19*	
STG, anterior	L	-52	4	-6	6.73	66
IFG opercularis	R	50	12	26	6.22	65
IFG triangularis	L	-44	28	10	6.06	45
Brainstem POTPT	L	-14	-26	-14	5.15	33
STG, mid	R	52	-4	-10	4.25	34
IFG triangularis	R	54	28	26	4.16	33
MTG, mid	R	68	-12	-20	3.85	13
Globus pallidus	R	20	2	-4	3.21	14
Putamen	R	22	4	12	2.72	10

L: left; R: right; MNI: Montreal neurological institute; MTG: middle temporal gyrus; STS: superior temporal sulcus; Cereb Crus: cerebellum crus of ansiform lobule; IFG: inferior frontal gyrus; STG: superior temporal gyrus; Brainstem POTPT: major brainstem motor tracts pathway of the parieto-occipito-temporo-pontine tract.

Supplementary Table 4: Activations, cluster size and coordinates for angry > neutral voices contrast, wholebrain voxel-wise *p*<.05 FDR correction, k>10.

		MNI coordinates				
Region label	Hemisphere	X	Y	Z	T value	Cluster size (voxels)
Precentral gyrus	R	60	8	26	12.77	11526
Postcentral gyrus	*R*	*-58*	*-14*	*18*	*10.91*	
Precentral gyrus	*R*	*52*	*2*	*46*	*10.88*	
STG, posterior	R	54	-34	12	9.75	84
STS, posterior	L	-64	-48	6	9.04	110
ITG, posterior	L	-46	-36	-16	8.71	85
Amygdala	L	-26	-6	-24	6.99	84
Supp Motor Area	R	8	6	58	6.36	47
Mid Frontal gyrus	L	-28	26	42	6.07	211
Mid Frontal gyrus	L	-40	36	22	5.70	309
Sup Frontal gyrus	L	-12	36	48	5.58	112
Cereb Nucl Fastigial	R	6	-56	-28	5.54	133
ACC	R	8	16	28	5.49	117
Globus Pallidus	R	18	-4	8	5.48	105
Cereb Crus 2	R	10	-84	-36	5.29	11
Globus Pallidus	L	-18	0	-4	5.07	44
Temporal pole	L	-58	10	-8	5.02	31
ACC	R	8	30	18	4.92	45
IFG triangularis	L	-50	28	10	4.72	14
Cereb Lob 8	L	-28	-58	-52	4.59	29
Supramarginal gyrus	L	-66	-22	28	4.59	13
IFG opercularis	L	-58	16	10	4.33	24
Sup Frontal gyrus	R	14	48	34	4.26	32
IFG triangularis	L	-42	20	8	4.17	36
Cereb Crus 2	L	-40	-74	-44	4.14	10
Putamen	L	-14	10	-10	4.09	27
Cereb Vermis 1-2	L	0	-42	-24	3.97	20
Cereb Lob 6	L	-30	-54	-38	3.96	26
Sup Frontal gyrus, medial	L	-8	48	36	3.91	33
ACC	L	-12	26	28	3.75	12
Cereb Crus 1	L	-40	-76	-26	3.74	18
STG, mid	L	-54	-10	-4	3.59	12
MTG, anterior	L	-62	-8	-18	3.28	20

L: left; R: right; MNI: Montreal neurological institute; STG: superior temporal gyrus; STS: superior temporal sulcus; ITG: inferior temporal gyrus; Supp Motor Area: supplementary motor area; Mid: middle; Sup: superior; Cereb Nucl Fastigial: fastigial nucleus of the cerebellum; ACC: anterior cingulate cortex; Cereb Crus: cerebellum crus of ansiform lobule; IFG: inferior frontal gyrus; Cereb Lob: cerebellum lobule; Cereb: cerebellum; MTG: middle temporal gyrus.

Supplementary Table 5: Activations, cluster size and coordinates for happy > neutral voices contrast, wholebrain voxel-wise $p<.05$ FDR correction, k>10.

		MNI coordinates				
Region label	Hemisphere	X	Y	Z	T value	Cluster size (voxels)
Precentral gyrus	R	54	-2	42	12.03	23099
STS, posterior	*R*	*62*	*-44*	*4*	*10.57*	
Rolandic operculum	*R*	*50*	*-18*	*20*	*9.89*	
Postcentral gyrus	L	-64	-12	20	7.05	594
Postcentral gyrus	*L*	*-66*	*-20*	*28*	*6.96*	
Mid Frontal gyrus	*L*	*-58*	*10*	*32*	*6.35*	
STG, anterior	L	-52	4	-6	6.39	59
STS, anterior	R	66	-12	-4	5.85	119
STS, anterior	*R*	*66*	*-4*	*-10*	*5.39*	
Sup Frontal gyrus	R	4	28	66	5.13	133
Mid Frontal gyrus	L	-38	38	28	4.99	60
STG, anterior	R	52	-4	-8	4.89	77
Temporal pole	*R*	*60*	*8*	*-4*	*3.87*	
IFG opercularis	L	-56	18	14	3.67	17
IFG triangularis	R	32	26	26	3.56	22
IFG opercularis	L	-44	20	32	3.39	13

L: left; R: right; MNI: Montreal neurological institute; STS: superior temporal sulcus; Mid: middle; STG: superior temporal gyrus; Sup: superior; IFG: inferior frontal gyrus.

Supplementary Table 6: Activations, cluster size and coordinates for angry & happy> neutral voices contrast, wholebrain voxel-wise *p*<.05 FDR correction, k>10.

		MNI coordinates				
Region label	Hemisphere	X	Y	Z	T value	Cluster size (voxels)
STS, posterior	R	62	-44	2	11.32	18286
Rolandic operculum	*R*	*50*	*-18*	*20*	*10.07*	
Mid Frontal gyrus	*R*	*44*	*0*	*54*	*9.89*	
Sup Frontal gyrus	L	-10	38	48	6.36	148
Mid Frontal gyrus	L	-28	36	16	6.11	348
STS, mid	L	-62	-32	-2	6.01	337
STS, mid	*L*	*-66*	*-22*	*-4*	*5.72*	
MTG, mid	*L*	*-66*	*-46*	*-6*	*4.95*	
Cereb Lob 9	L	-12	-56	-50	4.78	19
Insula	L	-40	-4	-8	4.33	18
Sup Temporal pole	L	-58	10	-8	4.18	40
Mid Frontal gyrus	L	-26	10	48	4.12	32
IFG triangularis	L	-50	28	10	4.10	68
IFG triangularis	R	46	22	14	4.06	24
Globus Pallidus	L	-18	0	0	3.86	18
IFG triangularis	L	-58	18	10	3.62	13
Sup Frontal gyrus, medial	L	-8	50	38	3.34	18

L: left; R: right; MNI: Montreal neurological institute; STS: superior temporal sulcus; Mid: middle; Sup: superior; MTG: middle temporal gyrus; Cereb Lob: cerebellum lobule; IFG: inferior frontal gyrus.

Supplementary Table 7: Activations, cluster size and coordinates for angry > neutral synthesized energy voices contrast, wholebrain voxel-wise *p*<.05 FDR correction, k>10.

		MNI coordinates				
Region label	Hemisphere	X	Y	Z	T value	Cluster size (voxels)
Precentral gyrus	R	60	8	26	7.99	226
Postcentral gyrus	L	-56	-12	18	7.57	397
MTG, posterior	R	60	-46	4	6.03	34
Insula	R	44	-10	4	5.81	216
STG, anterior	*R*	*56*	*-6*	*-4*	*5.23*	
STG, posterior	R	54	-34	12	5.58	25
STS, posterior	L	-62	-48	6	5.43	20
Hypothalamus	R	2	-4	-10	5.20	53
Amygdala	L	-26	-6	-24	5.12	28
MTG, anterior	R	66	-4	-16	4.89	24
Parahippocampal gyrus	R	26	-38	-16	4.72	151
Substantia nigra	L	-8	-28	-6	4.71	12
Parahippocampal gyrus	L	-24	-34	-4	4.04	17
Thalamus, LPN	L	-20	-16	10	3.65	10
Caudate head	R	4	10	-6	3.43	15
Putamen	L	-16	8	-8	3.40	10

L: left; R: right; MNI: Montreal neurological institute; MTG: middle temporal gyrus; STG: superior temporal gyrus; STS: superior temporal sulcus; LPN: lateral posterior nucleus.

Supplementary Table 8: Activations, cluster size and coordinates for happy > neutral synthesized energy voices contrast, *p*<.05 voxel-wise FDR correction, k>10.

Region label	Hemisphere	MNI coordinates X	Y	Z	T value	Cluster size (voxels)
Precentral gyrus	R	56	-2	42	7.96	229
STS, posterior	R	62	-44	4	6.82	486
STS, posterior	L	-64	-32	-4	6.30	377
Mid frontal gyrus	R	32	28	48	5.76	663
Putamen	R	28	-10	8	5.47	232
Parahippocampal gyrus	L	-22	-44	-4	5.19	454
IFG triangularis	L	-44	26	8	4.57	55
ACC	L	-4	12	38	4.45	153
Putamen	L	-28	-18	10	4.41	33
Amygdala	L	-26	-8	-22	4.20	63
Thalamus, VPLN	L	-18	-14	6	3.45	10
Caudate head	L	-8	20	0	3.35	10

L: left; R: right; MNI: Montreal neurological institute; STS: superior temporal sulcus; Mid: middle; IFG: inferior frontal gyrus; ACC: anterior cingulate cortex; VPLN: ventral posterior lateral nucleus.

Supplementary Table 9: Seed-to-seed functional connectivity (gPPI) for angry > neutral normal > f0 & energy synthesized voices contrast, *p*<.05 seed-level FDR correction, two-tailed.

Seed region label	Target region label	T value	pFDR
pSTG l	FO r	7.26	0.0006
GPe l	Cereb Lob X r	4.69	0.0470
STT l	POTPT r	5.71	0.0073
STT l	STT r	5.30	0.0077

FO: frontal operculum; GPe: external globus pallidus; pSTG: posterior superior temporal gyrus; STT: spinothalamic tract of the brainstem; POTPT: parieto-occipito-temporo-pontine tract of the brainstem; Cereb Lob: cerebellum lobule; l: left; r: right.

Supplementary Table 10: Seed-to-seed functional connectivity (gPPI) for happy > neutral normal > f0 & energy synthesized voices contrast, p<.05 seed-level FDR correction, two-tailed.

Seed region label	Target region label	T value	pFDR value
PaCC r	SubCC	-4.73	0.0442
pMTG l	COC r	4.70	0.0374
pMTG l	pSTG r	-4.45	0.0374
aSTG r	GPi r	4.70	0.0466
LL r	CST r	8.22	0.0001

PaCC: paracingulate cortex; SubCC: subcalcarine cortex; GPi: internal globus pallidus; COC: central operculum cortex; aSTG: anterior superior temporal gyrus; pSTG: posterior superior temporal gyrus; pMTG: posterior middle temporal gyrus; CST: corticospinal tract of the brainstem; LL: lateral lemniscus of the brainstem.; l: left; r: right.

Supplementary Table 11: Regions of interest included in the functional and/or effective connectivity analyses, all within the field of view of the acquired MRI volumes.

aal.IC r (Insular Cortex Right)

aal.IC l (Insular Cortex Left)

aal.SFG r (Superior Frontal Gyrus Right)

aal.SFG l (Superior Frontal Gyrus Left)

aal.MidFG r (Middle Frontal Gyrus Right)

aal.MidFG l (Middle Frontal Gyrus Left)

aal.PreCG r (Precentral Gyrus Right)

aal.PreCG l (Precentral Gyrus Left)

aal.aSTG r (Superior Temporal Gyrus, anterior division Right)

aal.aSTG l (Superior Temporal Gyrus, anterior division Left)

aal.pSTG r (Superior Temporal Gyrus, posterior division Right)

aal.pSTG l (Superior Temporal Gyrus, posterior division Left)

aal.aMTG r (Middle Temporal Gyrus, anterior division Right)

aal.aMTG l (Middle Temporal Gyrus, anterior division Left)

aal.pMTG r (Middle Temporal Gyrus, posterior division Right)

aal.pMTG l (Middle Temporal Gyrus, posterior division Left)

aal.toMTG r (Middle Temporal Gyrus, temporooccipital part Right)

aal.toMTG l (Middle Temporal Gyrus, temporooccipital part Left)

aal.aITG r (Inferior Temporal Gyrus, anterior division Right)

aal.aITG l (Inferior Temporal Gyrus, anterior division Left)

aal.pITG r (Inferior Temporal Gyrus, posterior division Right)

aal.pITG l (Inferior Temporal Gyrus, posterior division Left)

aal.toITG r (Inferior Temporal Gyrus, temporooccipital part Right)

aal.toITG l (Inferior Temporal Gyrus, temporooccipital part Left)

aal.PostCG r (Postcentral Gyrus Right)

aal.PostCG l (Postcentral Gyrus Left)

aal.aSMG r (Supramarginal Gyrus, anterior division Right)

aal.aSMG l (Supramarginal Gyrus, anterior division Left)

aal.pSMG r (Supramarginal Gyrus, posterior division Right)

aal.pSMG l (Supramarginal Gyrus, posterior division Left)

aal.AG r (Angular Gyrus Right)

aal.AG l (Angular Gyrus Left)

aal.SMA r (Juxtapositional Lobule Cortex - formerly Supplementary Motor Cortex- Right)

aal.SMA L(Juxtapositional Lobule Cortex - formerly Supplementary Motor Cortex- Left)

aal.SubCalC (Subcallosal Cortex)

aal.PaCiG r (Paracingulate Gyrus Right)

aal.PaCiG l (Paracingulate Gyrus Left)

aal.PC (Cingulate Gyrus, posterior division)

aal.aPaHC r (Parahippocampal Gyrus, anterior division Right)

aal.aPaHC l (Parahippocampal Gyrus, anterior division Left)

aal.pPaHC r (Parahippocampal Gyrus, posterior division Right)

aal.pPaHC l (Parahippocampal Gyrus, posterior division Left)

aal.aTFusC r (Temporal Fusiform Cortex, anterior division Right)

aal.aTFusC l (Temporal Fusiform Cortex, anterior division Left)

aal.pTFusC r (Temporal Fusiform Cortex, posterior division Right)

aal.pTFusC l (Temporal Fusiform Cortex, posterior division Left)

aal.FO r (Frontal Operculum Cortex Right)

aal.FO l (Frontal Operculum Cortex Left)

aal.CO r (Central Opercular Cortex Right)

aal.CO l (Central Opercular Cortex Left)

aal.PO r (Parietal Operculum Cortex Right)

aal.PO l (Parietal Operculum Cortex Left)

aal.PP r (Planum Polare Right)

aal.PP l (Planum Polare Left)

aal.HG r (Heschl's Gyrus Right)

aal.HG l (Heschl's Gyrus Left)

aal.PT r (Planum Temporale Right)

aal.PT l (Planum Temporale Left)

BG.1 left red nucleus

BG.2 right red nucleus

BG.3 left substantia nigra

BG.4 right substantia nigra

BG.5 left subthalamic nucleus

BG.6 right subthalamic nucleus

BG.7 left caudate

BG.8 right caudate

BG.9 left putamen

BG.10 right putamen

BG.11 left external globus pallidus

BG.12 right external globus pallidus

BG.13 left internal globus pallidus

BG.14 right internal globus pallidus

BG.15 left thalamus

BG.16 right thalamus

BG.17 left hippocampus

BG.18 right hippocampus

BG.19 left nucleus accumbens

BG.20 right nucleus accumbens

BG.21 left amygdala

BG.22 right amygdala

Cerebellum.1 Left_I_IV

Cerebellum.2 Right_I_IV

Cerebellum.3 Left_V

Cerebellum.4 Right_V

Cerebellum.5 Left_VI

Cerebellum.6 Vermis_VI

Cerebellum.7 Right_VI

Cerebellum.8 Left_CrusI

Cerebellum.9 Vermis_CrusI

Cerebellum.10 Right_CrusI

Cerebellum.11 Left_CrusII

Cerebellum.12 Vermis_CrusII

Cerebellum.13 Right_CrusII

Cerebellum.14 Left_VIIb

Cerebellum.15 Vermis_VIIb

Cerebellum.16 Right_VIIb

Cerebellum.17 Left_VIIIa

Cerebellum.18 Vermis_VIIIa

Cerebellum.19 Right_VIIIa

Cerebellum.20 Left_VIIIb

Cerebellum.21 Vermis_VIIIb

Cerebellum.22 Right_VIIIb

Cerebellum.23 Left_IX

Cerebellum.24 Vermis_IX

Cerebellum.25 Right_IX

Cerebellum.26 Left_X

Cerebellum.27 Vermis_X

Cerebellum.28 Right_X

Cerebellum.29 Left_Dentate nucleus

Cerebellum.30 Right_Dentate nucleus

Cerebellum.31 Left_Interposed nucleus

Cerebellum.32 Right_Interposed nucleus

Cerebellum.33 Left_Fastigial nucleus

Cerebellum.34 Right_Fastigial nucleus

Brainstem.CST Left (Corticospinal; motor)

Brainstem.CST Right (Corticospinal; motor)

Brainstem.FPT Left (Fronto-pontine; motor)

Brainstem.FPT Right (Fronto-pontine; motor)

Brainstem.ICPMC Left (Inferior Cerebellar ; cerebellar peduncles)

Brainstem.ICPMC Right (Inferior Cerebellar ; cerebellar peduncles)

Brainstem.ICPVC Left (Inferior Cerebellar; cerebellar peduncles)

Brainstem.ICPVC Right (Inferior Cerebellar; cerebellar peduncles)

Brainstem.LL Left (Lateral lemniscus; sensory tracts)

Brainstem.LL Right (Lateral lemniscus; sensory tracts)

Brainstem.MCP (Middle Cerebellar; cerebellar peduncles)

Brainstem.ML Left (Medial lemniscus; sensory tracts)

Brainstem.ML Right (Medial lemniscus; sensory tracts)

Brainstem.POTPT Left (Parieto-occipito-temporal; motor tracts)

Brainstem.POTPT Right (Parieto-occipito-temporal; motor tracts)

Brainstem.SCPCR Left (Superior Cerebellar; cerebellar peduncles)

Brainstem.SCPCR Right (Superior Cerebellar; cerebellar peduncles)

Brainstem.SCPCT Left (Superior Cerebellar; cerebellar peduncles)

Brainstem.SCPCT Right (Superior Cerebellar; cerebellar peduncles)

Brainstem.SCPSC Left (Superior Cerebellar; cerebellar peduncles)

Brainstem.SCPSC Right (Superior Cerebellar; cerebellar peduncles)

Brainstem.STT Left (Spinothalamic; sensory tracts)

Brainstem.STT (Spinothalamic; sensory tracts)

‘aal.’: Automated Anatomical Labelling atlas (NROI=58); ‘BG.’: Basal Ganglia atlas (NROI=22); ‘Cerebellum.’: Cerebellum atlas (NROI=34); ‘Brainstem.’: Brainstem atlas (NROI=23). For references, see Methods section.
